# Carotenoids from Starfish *Patiria pectinifera*: Therapeutic Activity in Models of Inflammatory Diseases

**DOI:** 10.3390/md21090470

**Published:** 2023-08-27

**Authors:** Aleksandr M. Popov, Emma P. Kozlovskaya, Anna A. Klimovich, Tatyana A. Rutckova, Aleksey I. Vakhrushev, Dmitry M. Hushpulian, Irina G. Gazaryan, Vyacheslav V. Makhankov, Oksana M. Son, Liudmila A. Tekutyeva

**Affiliations:** 1 G.B. Elyakov Pacific Institute of Bioorganic Chemistry, Far Eastern Branch of the Russian Academy of Science, 159 Prospect 100-letiya Vladivostoka, Vladivostok 690022, Russia; anka_zaraza13@mail.ru (A.A.K.); tanya1119@yandex.ru (T.A.R.); aivahr@mail.ru (A.I.V.);; 2 Faculty of Biology and Biotechnology, National Research University Higher School of Economics, 13/4 Myasnitskaya str., Moscow 117997, Russia; Hushpulian@gmail.com (D.M.H.); igazaryan@gmail.com (I.G.G.); 3 Bach Institute of Biochemistry, Federal Research Centre “Fundamentals of Biotechnology” of the Russian Academy of Sciences, Leninski prospect 33, Moscow 1190721, Russia; 4 Department of Chemical Enzymology, M.V. Lomonosov Moscow State University, Moscow 119991, Russia; 5 Department of Chemistry and Physical Sciences, Dyson College of Art and Sciences, Pace University, 861 Bedford Road, Pleasantville, NY 10570, USA; 6 School of Advanced Engineering Studies, Institute of Biotechnology, Bioengineering and Food Systems, Far Eastern Federal University, p. Ajax 10, Russky Island, Vladivostok 690922, Russia; oksana_son@bk.ru (O.M.S.); lat7777@mail.ru (L.A.T.); 7 ARNIKA, Territory of PDA Nadezhdinskaya, Volno-Nadezhdinskoye 692481, Russia

**Keywords:** carotenoids mixture (MC), starfish *Patiria pectinifera*, astaxanthin, therapeutic activity, inflammatory diseases

## Abstract

The carotenoids mixture (MC) isolated from the starfish *Patiria. pectinifera* contains more than 50% astaxanthin, 4–6% each zeaxanthine and lutein, and less pharmacologically active components such as free fatty acids and their glycerides. Astaxanthin, the major component of MC, belongs to the xanthophyll class of carotenoids, and is well known for its antioxidant properties. In this work, in vitro and in vivo studies on the biological activity of MC were carried out. The complex was shown to exhibit anti-inflammatory, anti-allergic and cancer-preventive activity, without any toxicity at a dose of 500 mg/kg. MC effectively improves the clinical picture of the disease progressing, as well as normalizing the cytokine profile and the antioxidant defense system in the in vivo animal models of inflammatory diseases, namely: skin carcinogenesis, allergic contact dermatitis (ACD) and systemic inflammation (SI). In the skin carcinogenesis induced by 7,12-dimethylbenzanthracene, the incidence of papillomas was decreased 1.5 times; 1% MC ointment form in allergic contact dermatitis showed an 80% reduced severity of pathomorphological skin manifestations. Obtained results show that MC from starfish *P. pectinifera* is an effective remedy for the treatment and prevention of inflammatory processes.

## 1. Introduction

Currently, natural biologically active substances (BASs), which have beneficial properties for human health, are of great interest to the consumer. A special place among these BASs is occupied by carotenoids (CRs), which are characterized by a high potential for protecting the body from a wide range of diseases, such as chronic inflammatory [1,2,3,4], neurodegenerative [5,6,7,8,9], cardiovascular [10,11,12,13], cancer [14,15,16,17], metabolic syndrome and diabetes [18,19,20,21], liver [22,23], skin [24,25,26] and eye [27] diseases, exercise-induced fatigue [21], infertility [28,29] and many other pathologies. Carotenoids are of particular interest as an adjuvant drug to reduce the cytokine storm, including those associated with COVID-19 [30,31]. It has been established that astaxanthin blocks oxidative damage to DNA, reduces the level of C-reactive protein and other biomarkers of inflammation [1,30,31,32]. Eating a diet rich in carotenoids has been shown to combat inflammation by increasing plasma concentrations of IFN-α2 and decreasing MIP-1β and TNF-α in human trials [1].

The CRs family is represented by more than 850 natural fat-soluble pigments, which are synthesized by algae, phytoplankton, plants, and some varieties of fungi and bacteria [33,34,35]. CRs are responsible for the coloration of various photosynthetic organisms [36]. Animals and humans are not able to synthesize CRs de novo; they obtain them with food [37,38,39] and actively use the metabolic capabilities of CRs [40]. For example, β-carotene is converted in the body to vitamin A, whereas lutein and zeaxanthin are macular pigments protecting the retina from damage by UV light [27,41].

As suggested [42,43], the antioxidant properties of CRs are the main mechanism by which they maintain and improve human health. Marine CRs (astaxanthin, fucoxanthin, β-carotene, lutein, as well as the rare siphonaxanthin, sioxanthin, and myxol) have recently demonstrated antioxidant properties in reducing markers of oxidative stress [1,44,45]. However, the interpretation of some results remains conflicting. It is difficult to explain CRs’ physiological effects only by their antioxidant activity; therefore more research is necessary to clarify the relationship between CRs’ action and ROS-mediated disorders. Over the past few years, genomic studies have focused on the unusual ability of CRs to regulate the expression of specific genes involved in cell metabolism [1,44,45].

To date, a large body of data has been collected in a number of epidemiological, interventional, and clinical studies, mainly from experiments with β-carotene, lycopene, astaxanthin, lutein, and zeaxanthin, which generally support the observation that an adequate intake of CR-rich fruits and vegetables or CR supplements can significantly reduce the risk of certain chronic diseases. The World Health Organization estimates that low fruit and vegetable intake is responsible for 31% of coronary heart disease, 11% of strokes, and 19% of gastrointestinal cancers worldwide.

Preparations based on echinoderm extracts have not yet been widely used in modern medicine, but are actively used in Chinese medicine for the prevention and treatment of a wide range of diseases. Increasingly, scientific works began to appear showing the effectiveness of the use of echinoderm extracts in the treatment of various diseases. In this regard, the research on the composition of metabolites of this type of animal is of great scientific interest [46,47].

*Patiria (=Asterina) pectinifera* [48] is an unpretentious species of starfish, widely distributed along the Western coast of the Pacific Ocean from the Sakhalin Island to the Yellow Sea. It is the most numerous species in the Peter the Great Bay (Sea of Japan).

**Figure 1:**
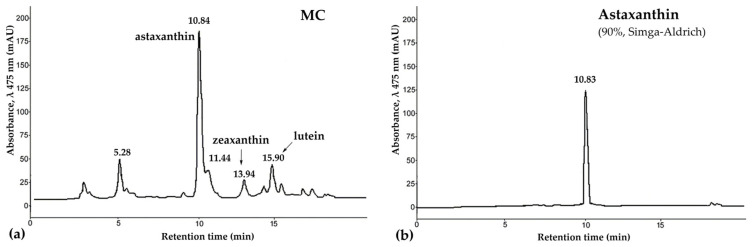
The high-performance liquid chromatography (HPLC) chromatogram of carotenoids mixture of starfish *P. pectinifera* (**a**) and sample of astaxanthin (90%, Sigma-Aldrich, St. Louis, MO, USA) (**b**). Chromatographic conditions: HPLC was carried out on a chromatograph LaChrom 2000 (Hitachi/Merck, Darmstadt, Germany) equipped with UV detector L-7400, pump L-7100, thermostat L-7300, integrator D-7500; column, Agilent Technologies Zorbax Eclipse XDB-C18, 3.5 µm (75 mm × 4.6 mm) with guard column Hypersil ODS, 5 µm (4.0 mm × 4.0 mm); detection, UV 475 nm; solvent, acetonitrile/water (85/15, *v*/*v*) with 1% glacial acetic acid; flow rate, 0.2 mL/min; thermostated at 30 °C. Abbreviations: MC—carotenoid mixture of starfish *P. pectinifera*.

The tissues of *P. pectinifera* contain a large amount of CRs, with the major component being astaxanthin [49,50,51]. Takashi Maoka et al. [51] almost completely, with the exception of 2%, identified carotenoids of the starfishes *A. pectinifera* and *Asterias amurensis*. They showed that starfish carotenoids are mainly astaxanthin derivatives. Our carotenoids mixture (MC) preparation contained 56% astaxanthin, 4% zeaxanthin, 6% lutein, and unidentified carotenoids (Figure 1). They can be used as raw materials for the production of new medicinal, cosmetic, and food products.

The goal of this work was to test the biological activity of a mixture of carotenoid pigments enriched in astaxanthin obtained from the starfish *P. pectinifera* according to the Russian patent [49] in a series of models of skin damage and systemic inflammatory diseases in vivo.

## 2. Results

### 2.1. Evaluation of Safety of Carotenoid mixture of Starfish P. pectinifera (MC)

To access the toxicity of the preparation, 12 healthy mice (6♂ and 6♀) of the intact group of animals were treated for 14 days. The changes in the body weight were in the normal mode, characteristic of healthy animals (Table 1). The general condition and behavior of the intact group met the criteria of the norm: they actively moved around the cage, drank water, and ate food. Food and water intake as well as hairline and mucous membranes remained normal.

The evaluation of the product’s safety in mice established that a single dose of the carotenoid complex orally and intraperitoneally had no toxic effect on the body weight compared to intact animals. The general condition of this group of experimental animals remained the same as the groups of healthy intact animals. Their behavior throughout the experiment did not differ from the behavior of healthy animals, i.e., they actively moved around the cage, drank water, and ate food. Feed and water intake as well as the condition of the hairline and mucous membranes remained normal after oral and intraperitoneal administration, with no local irritation observed, leading us to conclude on the safety of the MC use for therapeutic and prophylactic purposes.

### 2.2. The MC Effectiveness in Treatment of Carcinogenic and Allergic Skin Pathologies, and Systemic Inflammation (SI)

#### 2.2.1. Evaluation of Cancer-Preventive Activity of MC

**Figure 2:**
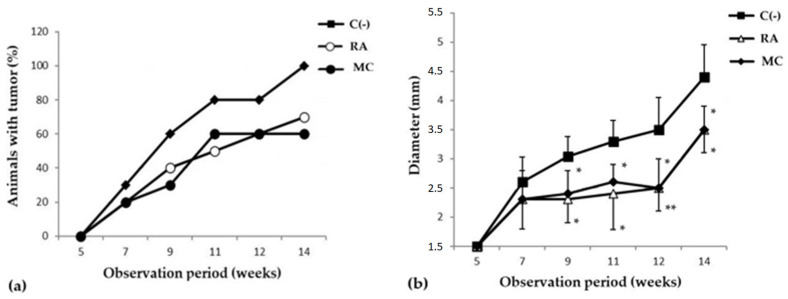
Dynamics of skin cancer incidence (**a**) and changes in the size of papillomas (**b**) (*y*-axis) in the experimental groups of animals. The data are presented from the moment the first changes appeared on the DMBA-treated animal skin areas (5 weeks after the start of the experiment). The results are presented as m ± SD (standard deviation) (n = 10), * *p* ≤ 0.05, ** *p* ≤ 0.01, compared with C(−) group. Abbreviations: C(−)—negative control; RA—rosmarinic acid (positive control); MC—carotenoids mixture of starfish *P. pectinifera*.

The study of the cancer-preventive activity of MC was carried out in a model of skin carcinogenesis induced by 7,12-dimethylbenzanthracene (DMBA). It was shown that during the period of active development of the oncological process, after frequent applications of DMBA, the groups of animals that received prophylactic oral administration of MC (10 mg/kg three times a week for 8 weeks) saw a decrease in the incidence of new tumor foci and in the growth intensity (Figure 2). It should be emphasized that MC was more effective than the reference substance, rosmarinic acid (RA), in inhibiting the formation of new papillomas (Table 2). So, in the MC C group, by the 11th week of the experiment, the formation of new tumor foci almost completely stopped, and by the 15th week, the number and size of papillomas were more than 1.5 times smaller than in the C(−) group (Figure 2b).

**Figure 3:**
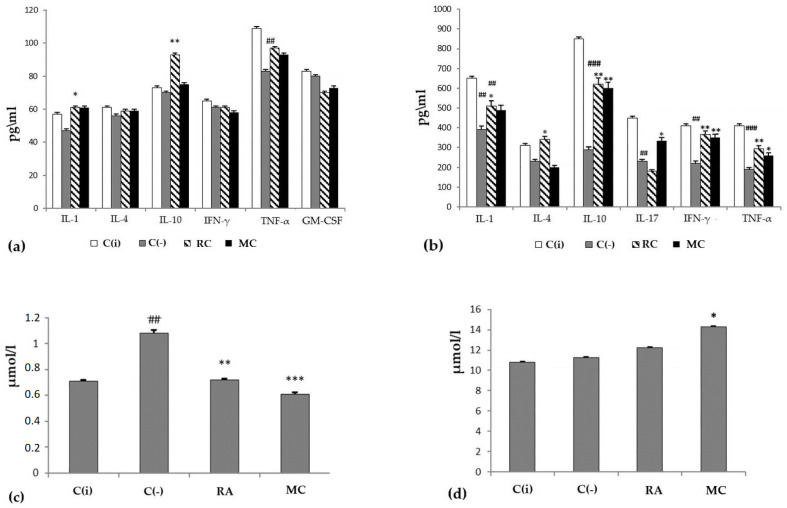
The profile of cytokines (*y*-axis, pg/mL) in blood serum (**a**) and skin homogenate (**b**), the level of malonic dialdehyde (MDA) (**c**) and total bilirubin (**d**) in the blood serum of animals (*y*-axis, µmol/L) at experimental modeling of skin carcinogenesis. The results are presented as m ± SD (standard deviation) (n = 10), * *p* ≤ 0.05, ** *p* ≤ 0.01, *** *p* ≤ 0.01, compared with the C(−) group; ## *p* ≤ 0.01, ### *p* ≤ 0.01, C(−) group compared to C(i) group. Abbreviations: C(−)—negative control; C(i)–intact control; RA—rosmarinic acid (positive control); MC—carotenoids mixture of starfish *P. pectinifera;* IL-1,4,10,17—interleukins-1,4,10,17; IFN-γ—interferon-gamma; TNF-α—tumor necrosis factor; GM-CSF—granulocyte–macrophage colony-stimulating factor.

Oxygenated carotenoids (astaxantin, zeaxanthin and lutein), the components of MC, are supposed to exert protective activity in oncological, allergic, and inflammatory processes mainly due to their ability to modulate the immune response [3,4,5,21,23,31,50,52]. When evaluating the functional activity of the immune system of experimental animals, it was shown that at the stage of progressive tumor growth in the C(−) group, onco-dependent immunosuppression was observed, which was expressed as a decrease in the level of IL-1, IL-17, IFN-γ, TNF-α, IL- 10, both systemically (blood serum) and locally (skin homogenate) (Figure 3a,b). Prophylactic oral intake of MC contributed to the stimulation of immunological surveillance, through an increase in the production of all of the above cytokines to the level close to the intact group, with the exception of IL-4. The data obtained indicate the ability of MC to modulate immunological reactions in skin lesions caused by DMBA and enhance the antitumor immune response.

**Figure 4:**
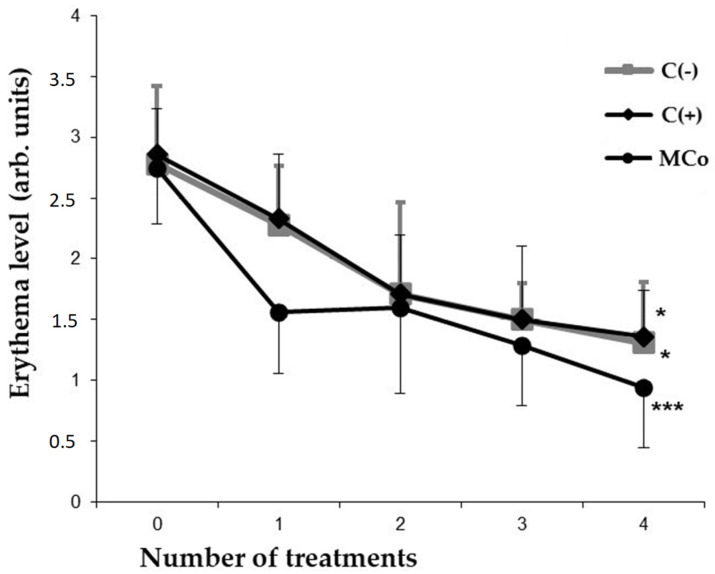
Indicators of the level of erythema (*y*-axis) caused by DNFB in different groups of animals after each day of treatment (*x*-axis). The results are presented as m ± SD (standard deviation) (*n* = 8), * *p* ≤ 0.05, *** *p* ≤ 0.01 (paired Student’s *t*-test). Abbreviations: DNFB—2,4-dinitrofluorobenzene; C(−)—negative control; C(+)—positive control ointment “Fucidin”; Mco—ointment, containing 1% carotenoids mixture of starfish *P. pectinifera*.

The state of the antioxidant system in the experimental animals was assessed by the concentration of malonic dialdehyde (MDA) (Figure 3c) in the blood serum. It was shown that in the C(−) group, the level of MDA was increased by 1.5 times compared to the group of intact animals. At the same time, MC turned out to be an effective corrector of this parameter. The general condition of the experimental animals was assessed by the level of bilirubin. The analysis showed that total bilirubin in the MC and RA groups was within the normal range (Figure 4d). Consequently, long-term administration of RA and MC did not exert a noticeable toxic effect on the animal organism.

Thus, in the groups of experimental animals treated with MC, a pronounced cancer-preventive effect was observed. MC is effective similarly to RA and improves the clinical picture of the disease progression, while normalizing the cytokine profile and the antioxidant defense system in the experimental animals.

#### 2.2.2. Evaluation of the MC Ointment Form in Allergic Contact Dermatitis (ACD)

**Figure 5:**
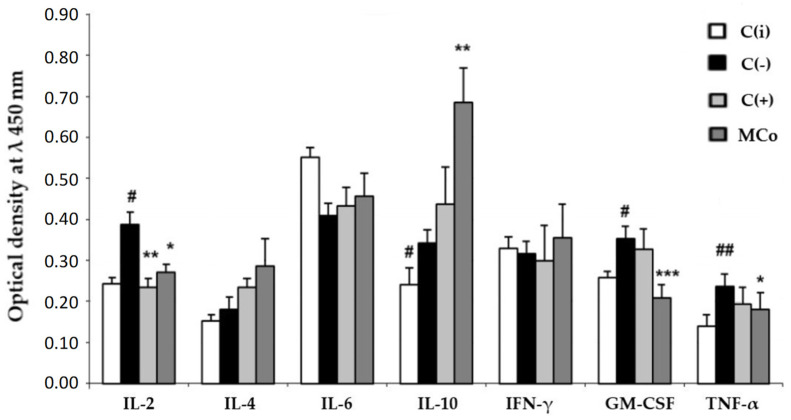
The level of cytokines in the blood serum (*y*-axis, optical density at λ 450 nm) in the experimental model of allergic contact dermatitis (ACD) induced by DNFB. Blood samples were obtained 24 h after the last application of the ointments. The results are presented as m ± SD (standard deviation) (*n* = 8), * *p* ≤ 0.05, ** *p* ≤ 0.01, *** *p* ≤ 0.01, compared with the C(−) group; # *p* ≤ 0.05, ## *p* ≤ 0.01, C(−) group versus C(i) group. Abbreviations: DNFB—2,4-dinitrofluorobenzene; C(−)—negative control; C(i)–intact control; C(+)—positive control ointment “Fucidin”; Mco—ointment containing 1% carotenoids mixture of starfish *P. pectinifera*; IL-2,4,6,10—interleukins–2,4,6,10; IFN-γ—interferon-gamma; TNF-α—tumor necrosis factor; GM-CSF—granulocyte–macrophage colony-stimulating factor.

In the case of administering a 1% MC ointment as an active compound, a significant restoration of the initial parameters of the skin and a decrease in the severity of external pathomorphological manifestations of ACD were observed. By the 4th day of treatment of experimental animals in the MC group, the index of reducing the severity of pathomorphological skin manifestations was 80% (Table 3). At the same time, the level of erythema decreased by approximately two times as compared to the C(−) group (Figure 4). It should be noted that the studied MC-based ointment was superior to the commercial anti-inflammatory drug “Fucidin” in terms of the effectiveness of the pharmacological action. MC acts as an effective corrector of the inflammatory process in ACD, the severity of which was assessed by the level of pro-inflammatory and anti-inflammatory cytokines in the blood serum by enzyme-linked immunosorbent assay (ELISA) (Figure 5).

It was found that the use of MC led to a decrease in the level of the main pro-inflammatory cytokines, involved in the pathogenesis of ACD, IL-2, TNF-α and GM-CSF, with a sharp increase in the main anti-inflammatory cytokines, IL-10 and IL-4, which may indicate the ability of MC components to enhance the anti-inflammatory response in ACD.

#### 2.2.3. Anti-Inflammatory Activity of MC in SI Model

**Figure 6:**
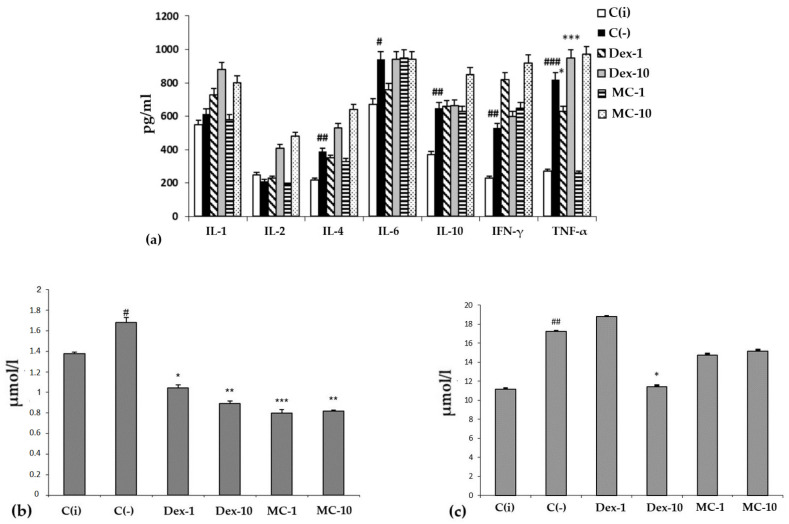
The content of cytokines (**a**) (*y*-axis, pg/mL), malonic dialdehyde (MDA) (**b**) and total bilirubin (**c**) (*y*-axis, in µmol/L) in the blood serum. The results are presented as m ± SD (standard deviation) (*n* = 6), * *p* ≤ 0.05, ** *p* ≤ 0.01, *** *p* ≤ 0.01, compared with the C(−) group; # *p* ≤ 0.05, ## *p* ≤ 0.01, ### *p* ≤ 0.01, C(−) group versus C(i) group. Abbreviations: C(−)—negative control, C(i) —intact control; Dex-1, Dex-10—“Dexamethasone” in doses of 1 and 10 mg/kg, respectively; MC-1, MC-10—oxygenated carotenoid complex of starfish *P. pectinifera* at doses of 1 and 10 mg/kg, respectively; MDA—malonic dialdehyde; IL-2,4,6,10—interleukins–2,4,6,10; IFN-γ—interferon-gamma; TNF-α—tumor necrosis factor.

When lipopolysaccharide (LPS) was administered to experimental animals, an increase in the serum content of pro-inflammatory cytokines, e.g., IL-1, -6, IFN-γ, and TNF-α, in the C (−) group was recorded (Figure 6a).

It is typical that the effect of MC and the reference drug “Dexamethasone” on the level of cytokines largely depends on the dose and route of administration of the drug. When administered orally, MC and Dexamethasone contributed to a sharp increase in the level of all tested cytokines compared to the C(i) and C(−) groups. However, with intraperitoneal administration, MC was more effective than Oftan-Dexamethasone in suppressing the production of key pro-inflammatory cytokines, IL-1 and TNF-α, bringing their level to that of the intact animals.

Thus, the ointment containing 1% MC of starfish *P. pectinifera* has high pharmacological prospects in the treatment of ACD and can be further used in various preclinical studies.

It should be noted that under conditions of SI, regardless of the dose and route of administration, the studied MC preparation had a protective effect on redox homeostasis, functional activity of the liver, biliary tract, and hematopoiesis, analyzed by the content of MDA and total bilirubin in blood serum in different experimental groups (Figure 6b,c). The conducted experimental studies in the SI model showed that MC has pronounced antioxidant and anti-inflammatory protective effects on the organism of experimental animals.

## 3. Discussion

One of the most promising strategies in the control of the incidence of oncological and inflammatory pathologies is chemoprophylaxis, which consists in the systematic use of prophylactic agents and functional nutrients that have a tumor-protective effect. At the same time, special attention is paid to compounds that have the ability to maintain redox homeostasis, exhibit an immunomodulatory effect, and have a positive effect on the functioning of various cell-signaling pathways with no side effects on the body of experimental animals. The results of our studies indicate that there is a direct correlation between the therapeutic use of MC of starfish *P. pectinifera* and a reduction in the risk of developing skin cancer (Figure 3, 1.5-fold reduction), and MC is more effective than rosmarinic acid (Table 2).

The Inhibition of the formation and growth of skin neoplasms observed with the use of MC may be the result not only from the antioxidant and immunomodulatory activity of carotenoids but also their ability to neutralize the carcinogenic effect of DMBA on epidermal cells, through the activation of the aryl hydrocarbon receptor (AhR). By stimulating the activity of AhR, they contribute to the stimulation of gene expression of biotransformation enzymes and the detoxification and elimination of xenobiotics, reducing the risk of accumulation of carcinogens in epithelial cells and thereby inhibiting the initial stages of carcinogenesis. It has been shown that AhR agonists are able to restrain neoplastic processes in the cell induced by chemical carcinogens, including DMBA [44,53,54,55]. The use of MC as prophylactic agent and functional nutrient can prevent the adverse effects of carcinogenic factors, maintain the optimal immunological status of the body and, thereby, reduce the risk of cancer. In addition to preventive action, MC has been shown to enhance the antitumor effect of the well-known cytostatic doxorubicin, which is widely used in chemotherapy, when they are used together [50]. In this regard, MC, after conducting preclinical trials, can expand the arsenal of complementary therapies in the treatment of oncological diseases.

Since carotenoids are considered not only as functional components of food, but also as active ingredients of cosmetic and cosmeceutical products, an important task is to evaluate their therapeutic effect in skin diseases, particularly in allergies. Allergic dermatoses are common inflammatory skin diseases with multifactorial etiology and complex pathogenesis. The most well-known diseases are psoriasis, contact dermatitis, eczema, chronic urticaria, and others. Pathogenesis includes primarily damage to the epidermal barrier as a result of an excessive inflammatory response from the immune system, in response to the action of various irritants (mechanical damage to the skin, contact with chemical, food, household, and other allergens). Currently widely used antiallergic drugs (glucocorticoids, antihistamines, mast cell stabilizers, and immunosuppressants) have a strong antiallergic effect. However, due to the narrow range of pharmacological action, none of these drugs are universal for the treatment of all types of skin allergies, and often they require long-term use, which can lead to a number of side effects: central nervous system depression, impaired carbohydrate and lipid metabolism, myasthenia gravis, osteoporosis, decreased resistance to infections [55]. In this regard, it is necessary to develop universal effective drugs that, along with antiallergic activity, can also inhibit inflammatory reactions and stimulate reparative processes in the epidermis. The therapeutic effect of the MC–-based ointment was seen in a noticeable improvement (80% skin restoration effect, Table 3) in pathomorphological and biochemical parameters of the disease, i.e., a significant decrease in the level of erythema and correction of the cytokine profile. Previously, Meephansan et al. studied the effect of astaxanthin on skin wound healing [56]. Full-thickness skin wounds were created in 36 healthy female mice, which were divided into a control group and a group treated with 78.9 mg locally of astaxanthin twice a day for 15 days. Astaxanthin-treated wounds showed marked narrowing by day 3 of treatment and complete wound closure by day 9, while wounds in control mice showed only partial epithelialization and still had scabs. Biological markers of wound healing, including Col1A1 and bFGF, were significantly elevated in the astaxanthin group from day 1. The results show that astaxanthin is an effective wound-healing compound. Chow et al. showed that the astaxanthin-enriched extract (EAE) from H. pluvialis increased the expression of certain proteins that promote cell proliferation, had a higher cell growth capacity than doxycycline, and was able to proliferate more collagen than the control group over seven days [57]. This indicates that it is the best alternative for collagen production.

Immunological blood testing (Figure 6) showed that MC acts as an effective corrector of the inflammatory process in ACD, its use led to a decrease in the level of the main pro-inflammatory cytokines involved in the pathogenesis of ACD: IL-2, TNF-α and GM-CSF. It should be noted that under the action of MC, the content of the main anti-inflammatory cytokines sharply increased: IL-10 and IL-4. This fact may indicate the ability of MC to enhance the anti-inflammatory response in ACD. It can be assumed that these carotenoids have an anti-inflammatory effect, preventing the synthesis of endogenous inhibitors of matrix metalloproteinases. The latter play a central role in the metabolism of connective tissue proteins, as they promote tissue renewal and are essential for skin repair and removal of inflammatory agents [58]. In 2010, Park et al. conducted the first comprehensive study investigating the effects of dietary ASX on modulating immune response, oxidative status, and inflammation in young healthy adult female subjects [59]. After eight weeks of supplementation, ASX enhanced both cell-mediated and humoral immune responses, including T cell and B cell mitogen-induced lymphocyte proliferation, NK cell cytotoxic activity, and IL-6 production. ASX did not affect plasma C-reactive protein concentrations, but levels of 8-hydroxy-2’-deoxyguanosine (8-oHdG) (a biomarker of DNA damage) were significantly lower in the higher-dose ASX group.

The study on the effect of MC on the immunological status of animals in the model of skin carcinogenesis, ACD and SI, indicates that it is an anti-inflammatory agent when administered intraperitoneally, but not orally. Altogether, the obtained results show that MC from sea star *P. pectinifera* is an effective remedy for the treatment and prevention of inflammatory processes.

## 4. Materials and Methods

### 4.1. Preparations

The method for obtaining an MC from starfish *P. pectinifera* includes the extraction of raw materials with 96% alcohol with the addition of food acid and ascorbic acid as a food antioxidant, column chromatography on a hydrophobic sorbent Polychrome-1, balanced with 20–30% alcohol. The column with adsorbed carotenoids is washed with alcohol in a gradient of 30→50%. The mixture of carotenoids is eluted with 60–65% alcohol. The eluate is evaporated in vacuum at 40–60 °C. Additional purification of the MC from phospholipids carried out by dissolving the concentrate in 96% alcohol, settling solution for 24–48 h at (−18 °C), centrifugation and subsequent evaporation in vacuum at 40–60 °C [49].

The MC composition was determined by high-performance liquid chromatography, UV and IR spectroscopy (Figure 1).

For intraperitoneal and oral injections, MC were weighed according to the dose used and prepared in aqueous, water–alcohol (100:1, *v*/*v*) or aqueous solutions with DMSO (Sigma-Aldrich, St. Louis, MO, USA) (10:1, *v*/*v*). Ointment for external use was prepared on a lanolin–Vaseline basis (1:3 by weight): 1 g of MC (1%) was added to 100 g of the ointment base.

### 4.2. Animals

CBA, CD-1, and BALB/c mice (20 ± 2 g) were used as a test system for studying the activity of natural substances. The experiments were performed on animals purchased from the laboratory animal nursery “Pushchino” and bred in the vivarium of the Pacific Institute of Bioorganic Chemistry of the Far Eastern Branch of the Russian Academy of Sciences (PIBOC FEB RAS) (certificate available).

The animals were kept in accordance with the GOST 33216-2014 “Guidelines for accommodation and care of animals. Species-specific provisions for laboratory rodents and rabbits”. After the experiment was completed, they were subjected to euthanasia. Studies using experimental animals were carried out in accordance with the GOST 33044-2014 “Principles of good laboratory practice” and “Guidelines for conducting preclinical studies of drugs”, edited by Mironov et al. [60]. All experiments were approved by the Ethical Committee for Animal Research of the PIBOC FEB RAS, protocol code 08/19, date of approval 27 March 2019.

### 4.3. Safety Assessment of MC

The safety assessment of MC was carried out in accordance with the guidelines for studying the toxic effects of pharmacological substances [61]. The experiments were performed on female and male mice of the CD-1 line (24 ± 2 g). Experimental groups of animals: 36 mice were used in the experiment, including 18 females and 18 males. Animals were randomized into 3 groups. The experimental group included 12 animals, 6 males and 6 females, who were administered MC orally and intraperitoneally in a water–alcohol solution in a volume of 0.5 mL. The control group of intact animals, 6 females and 6 males, were injected with distilled water. The control negative group, 6 females and 6 males, consisted of the animals that were injected with a water–alcohol solution (solvent).

### 4.4. Murine Model of Skin Carcinogenesis

The study of the cancer-preventive activity of MC was carried out on a model of skin carcinogenesis induced by 7,12-dimethylbenzanthracene (DMBA) (Sigma-Aldrich, St. Louis, MO, USA), which was dissolved in benzene and applied to the shaved interscapular region of experimental animals at a dose of 10 μg per mouse, 3 times a week for 8 weeks. The cancer-protective effect of the studied substances was assessed by their effect on pathomorphological changes in tissues: changes in the number of animals with a tumor, size, number of tumor formations (diameter, mm) and latent period (the period between the appearance of the first signs of oncogenesis until the appearance in the group of 50% of animals with tumor). Rosmarinic acid (RA) (Sigma-Aldrich, St. Louis, MO, USA) was used as a positive control.

### 4.5. Murine model of Experimental Dermatitis

Experimental ACD was reproduced using the obligate allergen 2,4-dinitrofluorobenzene (DNFB) (Sigma-Aldrich, St. Louis, MO, USA) in the form of a 0.5% oil-acetone mixture (acetone: olive oil, 4:1, by volume) for sensitization (applied once on shaved area of the peritoneum) and a 0.2% mixture to obtain an extensive disease (double application to the inner and outer surface of the ear). The effect of ointment preparations on the degree of healing of the affected areas and the level of erythema (redness and induration of the area, inflamed tissues with hyperemia, lichenification of the skin and the formation of a superficial hemorrhagic crust and areas of necrosis) was assessed. Commercial ointment “Fucidin” (Leo Laboratoris Limited, Dublin, Ireland) was used as a reference drug.

### 4.6. Murine Model of Inflammation

SI was induced by LPS from *Escherichia coli* (Sigma-Aldrich, St. Louis, MO, USA) at a dose of 0.1 mg/kg. The anti-inflammatory commercial drug Oftan Dexamethasone (Santen AO, Tampere, Finland) was used as a positive control. The test substances were administered to the animals 1 h before LPS induction. An hour and a half after SW induction, blood samples were taken for immunological and biochemical analyses.

### 4.7. Immunological and Biochemical Parameters Studies

The functional state of the immune system was assessed by determining the level of cytokines by ELISA using diagnostic kits (BD Bioscience OptEIA, Bergen, NJ, USA).

The severity of free-radical processes in pathological processes was determined by the content of TBA-reactive products (MDA) in blood plasma, by reaction with thiobarbituric acid (TBA) (Sigma-Aldrich, St. Louis, MO, USA).

To assess the effect of the studied dietary supplements on the general condition of the body, an analysis was made for total bilirubin using the Novogluk-KM kit (Vector-Best, Novosibirsk, Russia). The optical density of the samples was measured using an ELS 808 iu plate reader (BioTek, Winooski, VT, USA) at a wavelength of 450 nm.

### 4.8. Statistics

Statistical and graphical processing of experimental data was carried out using the statistical package Microsoft Excel Office version number 12.0. The obtained values were expressed as mean ± SD (standard deviation). The significance of differences was determined using a parametric Student’s *t*-test. Differences at *p* < 0.05 were taken as significant.

## 5. Conclusions

Evaluation of the functional activity of the antioxidant defense system and the general state of the body of experimental animals, when modeling the SI, showed that MC in all studied doses and routes had a protective effect on redox homeostasis and the functional activity of the liver, biliary tract, and hematopoiesis, and had no toxic side effects.

The results of the study of the effect of MC on the immunological status of animals, when modeling skin carcinogenesis, ACD, and SI, testify in favor of the conclusion that MC manifests itself as a local, and oral, anti-inflammatory agent. Namely, MC reduces the excessive immune response by direct action on the focus of inflammation (ip injection in SI, applications on the inflamed skin area in ACD), and at the same time has an immunostimulatory effect when administered enterally (oral administration for skin carcinogenesis and SI). Therefore, the selection of the route of administration of the oxygenated carotenoid products largely determines their physiological effect on the body. Based on the results obtained, it can be concluded that the *P. pectinifera* starfish MC is a promising candidate for the treatment and prevention of inflammatory processes. This finding creates the background for a comprehensive study of MC biopharmaceutical properties and its optimization for medical and cosmetic practice.

## Figures and Tables

**Table 1:** Weight (g) of healthy intact mice, mice after a single intraperitoneal or oral administration of a water–alcohol solution (solvent) and MC.

No., Gender	Intact Animals		Water-Alcohol Solution (Solvent) in a Volume of 0.5 mL		MC at a Dose of 500 mg/kg.	
	First Day	On the 14th Day	Before Administration	On the 14th Day	Before Administration	On the 14th Day
			IP injection			
1♂	23.5	30.2	22.6	30.6	23.0	31.1
2♂	23.4	31.1	23.4	30.1	23.8	31.8
3♂	22.8	31.6	22.8	31.1	22.3	31.2
1♀	21.3	30.8	22.1	30.9	22.4	29.8
2♀	23.4	31.1	23.2	32.1	23.0	31.1
3♀	23.2	31.6	22.4	31.3	22.6	30.3
			Oral administration			
1♂	22.4	30.9	22.2	31.3	23.6	29.9
2♂	22.9	31.1	23.4	31.1	23.2	30.4
3♂	23.3	31.1	23.8	31.8	22.3	30.1
1♀	22.9	30.4	22.3	30.4	23.3	30.4
2♀	23.5	31.8	23.8	32.1	23.8	32.1
3♀	22.4	33.1	23.4	32.3	22.8	31.3

Abbreviations: MC—carotenoids mixture of starfish *P. pectinifera;* IP—Intraperitoneally administration; ♂—male; ♀—female.

**Table 2:** Chemopreventive effect of MC in the experimental model of skin carcinogenesis.

Group	Number of Animals with Tumor, %	Number of Tumors	Average Tumor Diameter, mm	Latent Period, Week
Intact	-	-	-	-
C(−)	100	27	4.4 ± 1.6	4.2 ± 2.9
RA	70	15	3.5 ± 0.9	4.6 ± 1.1
MC	60	13	3.5 ± 1.01	4.3 ± 2.7

Abbreviations: C(−)—negative control; RA—rosmarinic acid (positive control); MC—carotenoids mixture of starfish *P. pectinifera.*

**Table 3:** Effect of MC ointment on the process of restoration of skin integuments affected by DNFB.

Group Animals	1 Days	2 Days	4 Days
	Healing, %, (m ± σ)		
C(−)	-	-	-
Fucidin	8 ± 2.5	20 ± 3.5	24 ± 2.3
MC, 1%	43 ± 2.9	40 ± 2.6	80 ± 1.3

Abbreviations: C(−)—negative control without treatment; Fucidin—positive control treatment ointment “Fucidin”; MCo—ointment, containing 1% carotenoids mixture of starfish *P. pectinifera*.

## Data Availability

Not applicable.
